# Concurrent chemo-radiotherapy with proton therapy: reduced toxicity with comparable oncological outcomes vs photon chemo-radiotherapy

**DOI:** 10.1038/s41416-020-0919-2

**Published:** 2020-06-18

**Authors:** Brian C. Baumann, Dennis E. Hallahan, Jeff M. Michalski, Carlos A. Perez, James M. Metz

**Affiliations:** 1 Department of Radiation Oncology, Washington University School of Medicine, St. Louis, MO USA; 2 Department of Radiation Oncology, University of Pennsylvania Perelman School of Medicine, Philadelphia, PA USA

**Keywords:** Cancer, Radiotherapy

## Abstract

Concurrent chemo-radiotherapy is a commonly employed curative treatment approach for locally advanced cancers but is associated with considerable morbidity. Chemo-radiotherapy using proton therapy may be able to reduce side effects of treatment and improve efficacy, but this remains an area of controversy and data are relatively limited. We comment on recently published studies and discuss future directions for proton therapy.

## Main

Concurrent chemo-radiotherapy is a commonly employed curative treatment approach for locally advanced cancers. Chemo-radiotherapy is used as definitive treatment or in the pre- or post-operative setting for head and neck, lung, GI, gynaecological and brain cancers among others.^1^ The morbidity of concurrent chemo-radiotherapy can be considerable,^2^ in spite of advances in radiotherapy techniques, improved systemic therapy and supportive care strategies. Most concurrent chemo-radiotherapy patients in the UK, Europe and North America are treated with X-ray radiation, which irradiates the target but deposits radiation both as the beams enter and exit the body. Proton therapy has generated considerable interest as an alternative radiation therapy modality that may reduce toxicity, as radiation can be delivered to the target without exit dose passing through tissues beyond the target.^3^ For patients treated with concurrent chemo-radiotherapy, proton therapy is particularly attractive because it allows us to reduce the volume of adjacent normal tissue exposed to both high-dose chemotherapy and radiotherapy, which may reduce side effects. While proton therapy may be able to lower side effects, the cost of proton therapy is considerably higher due to the large capital expenditure and higher maintenance costs for a proton facility. Research to determine if reductions in toxicity can justify the higher upfront cost of proton treatments and whether proton therapy can improve oncological outcomes is ongoing.^1^

Early results from single-institution trials randomising to proton vs photon chemo-radiotherapy are now available, but results are preliminary, and it is difficult to draw firm conclusions based on the available data. The first randomised clinical trial comparing proton vs photon chemo-radiotherapy was reported in 2017 in non-small-cell lung cancer (*n* = 149).^4^ The authors found no difference in the primary endpoint of grade 3 radiation pneumonitis with no difference in 12 month local control. The study did not report on rates of other toxicities, so it is unclear if proton therapy was associated with lower overall rates of grade ≥3 adverse events. More recently, preliminary results from a Phase 2 trial investigating proton vs photon chemo-radiotherapy in oesophageal cancer were presented at the 2019 ASTRO Annual Meeting. This study found that the total toxicity burden, defined as a severity-weighted sum over 11 specific severe adverse events, was significantly lower in the proton cohort, with no differences in progression-free survival after a median follow-up of 11 months in the 105 evaluable patients.^5^ Trials of proton vs photon chemo-radiotherapy are ongoing in head and neck cancer.

Interest in the comparative effectiveness of proton vs photon chemo-radiotherapy has been growing. In the US, there are several actively accruing co-operative-group-randomised trials investigating this question, including NRG BN-005 in glioma, NRG GI-006 in oesophageal cancer and RTOG 1308 in lung cancer. It will take several years for these trials to accrue, and enrolment has been challenging due to the limited number of proton centres in the US and challenges getting US insurers to cover proton treatments.^6^

Our group recently reported results from the largest series of proton vs photon chemo-radiotherapy patients who were prospectively followed for adverse events and oncological outcomes.^1^ In this series, 1483 adult patients with locally advanced, non-metastatic cancer were treated with proton vs. photon chemo-radiotherapy with curative intent from 2011–2016 at the University of Pennsylvania. Three hundred ninety-one received proton therapy and 1092 received photon therapy. The primary endpoint was 90-day adverse events associated with unplanned hospitalisations (CTCAEv4 grade ≥3). Secondary endpoints included ECOG performance status decline during treatment, 90-day adverse events of at least grade 2 that limit instrumental activities of daily living and disease-free survival and overall survival. Propensity analysis was performed to account for measured confounders. Proton therapy patients were significantly older with more comorbidities, owing to the fact that the government-sponsored healthcare for retirees in the US (Medicare) generally covers proton therapy, whereas private insurers for working-age patients generally do not. Grade ≥3 adverse events occurred in 28% of the photon cohort and 12% of the proton cohort (*P* < 0.001). On propensity score weighted analyses, proton chemo-radiotherapy was associated with significantly lower relative risk (RR) of 90-day grade ≥3 adverse events (RR 0.31; 95% CI [confidence interval], 0.15–0.66, *P* = 0.002), 90-day grade ≥2 adverse events (RR 0.78; 95% CI 0.65–0.93, *P* = 0.006) and decline in performance status (RR 0.51; 95% CI, 0.37–0.71; *P* < 0.001). There were no statistically significant differences in disease-free or overall survival, although the adjusted survival curves slightly favoured proton therapy. The fact that proton therapy was not associated with improved survival was not surprising since the overall approach to radiotherapy and chemotherapy at the University of Pennsylvania did not vary based on whether patients were treated with proton or photon therapy.

We think this study finding a significant improvement in severe adverse events with proton chemo-radiotherapy has several important implications for future research endeavours. The results are exciting as they at least raise the possibility that the higher upfront cost of proton therapy may be offset by savings from reduced hospitalisations and improved productivity from cancer patients, their families and caregivers. In a nationalised health system such as the NHS, the potential for proton therapy to reduce the total cost of care is arguably more appealing to payers than in the US system of employer-based private insurance as private insurers are less likely to recoup all of the potential downstream cost savings associated with proton therapy because patients typically change health insurers when they change jobs. More work is certainly needed to investigate the cost effectiveness of proton chemo-radiotherapy. The study findings showing significantly reduced toxicity with proton therapy also present an opportunity to improve survival outcomes for chemo-radiotherapy patients—by exploring treatment intensification trials, such as dose-escalated radiotherapy and/or dose-intensified systemic therapy as well as trials testing novel systemic therapies that were felt to be too toxic in combination with conventional radiotherapy. Lastly, proton therapy offers an opportunity to improve outcomes for older, sicker patients who could more readily tolerate chemo-radiotherapy if it is delivered with proton therapy. These patients are often not offered combined modality treatments in current practice and are traditionally excluded from clinical trials of combined modality therapy.^1^

The main limitation of the study is that patient allocation to therapy was non-randomised. We performed propensity analysis to adjust for measured confounders using a robust and complete database with over 130 clinical, demographic, and treatment-related variables. We explored the effect of unmeasured confounders and found that a very large imbalance in an unmeasured confounder would be needed to change the overall study results. In a subsequent analysis, we investigated demographic factors in the two cohorts to see if the cohort with proton-accepting insurance was somehow more privileged. We found that there was no difference in the relative risk of grade ≥3 adverse events for patients treated with photon chemo-radiotherapy who had proton-accepting insurance vs those with non-proton-accepting insurance.^7^

Prospective clinical trials of proton vs photon chemo-radiotherapy are warranted to validate our findings. We think the current data on the use of proton chemo-radiotherapy is sufficiently compelling that insurers should reconsider their coverage policies for proton therapy and should strongly consider covering proton therapy on randomised trials of photon vs proton therapy. The UK can play an important role in evaluating the role of proton therapy as part of concurrent chemo-radiotherapy, with the opening of proton centres in Manchester and London, and the UK’s excellence in running high-quality oncology trials.^8^

## Data Availability

Not applicable.
